# Concordance and generalization of an AI algorithm with real-world clinical data in the pre-omicron and omicron era

**DOI:** 10.1016/j.heliyon.2024.e25410

**Published:** 2024-02-02

**Authors:** Gulsen Yilmaz, Sevilay Sezer, Aliye Bastug, Vivek Singh, Raj Gopalan, Omer Aydos, Busra Yuce Ozturk, Derya Gokcinar, Ali Kamen, Jamie Gramz, Hurrem Bodur, Filiz Akbiyik

**Affiliations:** a Department of Medical Biochemistry, Ankara Yıldırım Beyazıt University, Ankara, Turkey; b Department of Medical Biochemistry, Ministry of Health, Ankara Bilkent City Hospital, Ankara, Turkey; c Department of Infectious Disease and Clinical Microbiology, Health Science University of Turkey, Gulhane Medical School, Ankara City Hospital, Ankara, Turkey; d Siemens Healthineers, Digital Technology and Innovation, Princeton, NJ, USA; e Siemens Healthineers, Diagnostics, Tarrytown, NY, USA; f Department of Infectious Disease and Clinical Microbiology, Ankara Bilkent City Hospital, Ankara, Turkey; g Department of Anesthesiology and Reanimation, Health Science University Turkey, Ankara Bilkent City Hospital, Ankara, Turkey; h Ankara Bilkent City Hospital Laboratory, Medical Director, Siemens Healthineers, Ankara, Turkey

**Keywords:** COVID-19, Algorithms, Predictive value of tests, Disease severity, Clinical laboratory tests

## Abstract

All viruses, including SARS-CoV-2, the virus responsible for COVID-19, continue to evolve, which can lead to new variants. The objective of this study is to assess the agreement between real-world clinical data and an algorithm that utilizes laboratory markers and age to predict the progression of disease severity in COVID-19 patients during the pre-Omicron and Omicron variant periods. The study evaluated the performance of a deep learning (DL) algorithm in predicting disease severity scores for COVID-19 patients using data from the USA, Spain, and Turkey (Ankara City Hospital (ACH) data set). The algorithm was developed and validated using pre-Omicron era data and was tested on both pre-Omicron and Omicron-era data. The predictions were compared to the actual clinical outcomes using a multidisciplinary approach. The concordance index values for all datasets ranged from 0.71 to 0.81. In the ACH cohort, a negative predictive value (NPV) of 0.78 or higher was observed for severe patients in both the pre-Omicron and Omicron eras, which is consistent with the algorithm's performance in the development cohort.

## 1 Introduction

The World Health Organization designated Coronavirus disease-2019 (COVID-19) a pandemic in March 2020 after the epidemic disease first appeared in China in December 2019 [1]. The Severe Acute Respiratory Syndrome-Coronavirus-2 (SARS-CoV-2) virus has been responsible for an estimated 591 million cases of global health problems, including over 6 million deaths [1]. SARS-COV-2 infection can have a variety of symptoms ranging in severity from influenza to death. About 15 % of cases caused by human coronavirus strains are the common cold in its mild form [2]. To better manage the clinical situation, it will be helpful to identify specific laboratory indicators that could differentiate between severe and non-severe cases or between the high and low risk of death [3].

Improving the early screening, diagnosis, and prognosis of the disease are critical steps for reducing COVID-19 deaths during this pandemic. Various studies have been carried out using Artificial Intelligence (AI) approaches to optimize these procedures in terms of quality, accuracy, and, most importantly, time in clinical settings since the WHO declared the COVID-19 outbreak a pandemic [[4], [5], [6], [7], [8]]. On Nov 26, 2021, a novel variant of concerns (VoCs) for SARS-CoV-2, Omicron, was documented about 23 months after the initial reported case of COVID-19 [9]. In comparison to earlier VoCs, the Omicron (B.1.1.529) variant displayed a faster doubling time than Delta, a longer infectiousness period, and greater rates of reinfection [10]. Omicron's key concerns are if it is more severe or contagious than other VoCs. Numerous studies have used machine learning to diagnose and predict the prognosis of different COVID-19 variants [9,[11], [12], [13]]. As a result, the question of whether AI algorithms trained to predict COVID-19 severity using pre-Omicron variant data remains valid for use during the Omicron variant era.

The use of AI, which can be defined simply as the imitation of human intelligence, is increasing in laboratory medicine, similar to other branches of medicine. With the COVID-19 pandemic, the need for AI technologies to control the expanding burden in healthcare has become even more obvious. AI applications have been used in many areas, from estimating the epidemiological course of the disease to the development of different diagnostic tools and from modeling the virus to treatment algorithms to control the pandemic. A number of models have been created for the screening, diagnosis, and prognosis of COVID-19 with AI tools developed using clinical laboratory results obtained from patients with SARS-CoV-2 infection [[5], [6], [7]]. AI techniques using deep learning (DL) methods have shown great success in the field of medical imaging due to the advanced feature extraction capability of DL. Aside from the field of medical imaging, numerous studies have used AI techniques to screen, diagnose, and predict the prognosis of COVID-19 using clinical, laboratory, and demographic data [[11], [12], [13], [14], [15]]. Despite these advancements, we have yet to see a practical system that could be used universally with evidence of generalizability to aid in the early identification of patients who develop severe clinical outcomes. Along with all these developments, some limitations regarding the use of AI were also seen during routine clinical practice. It has been revealed that there are deficiencies in accessing appropriate data, validation of developed models, and multidisciplinary approach [5].

A prognostic DL model was developed and externally validated by Siemens Healthineers (Siemens Healthineers, Erlangen, Germany), the working partner of Ankara City Hospital Medical Biochemistry Laboratory services, using data from hospitals in the US and Spain, based on laboratory tests to predict the need for care, risk of death, severity assessment or length of stay in the hospital [16]. The schema of the used method (i.e., deep profiler) is based on deriving a patient fingerprint from various demographic, clinical, and laboratory parameters and utilizing it to predict severity scores. Deep profiler consists of three main parts (i.e., networks): an encoder network for extracting prominent features represented in a latent space, which is also referred to as the patient fingerprint, a decoder network for reconstructing the input data to ensure data fidelity of the latent feature representation, and finally a severity classifier network, which is trained to estimate the severity score [17]. The prognosis was predicted using this model based on commonly assessed laboratory characteristics consisting of clinical chemistry, complete blood count (CBC), and coagulation tests, which are requested and performed within the first 72 h of admission. However, the effectiveness of this previously developed algorithm has not been determined by comparing its predictions of severity to the outcomes of actual clinical data collected in Turkey.

This retrospective cohort study's objective was to assess the consistency between prognostic information predicted by the previously created and validated DL algorithm and the actual clinical course of COVID-19 patients with varying disease severity before and after the Omicron variant with a multidisciplinary approach under the same laboratory conditions. In this way, it is to evaluate whether the DL algorithm can be generalized as a reliable tool for prognosis prediction.

## 2 Materials and methods

COVID-19 patients who were hospitalized to the Infectious Diseases Clinic with confirmed diagnosis by detecting SARS-CoV-2 RNA in oro-nasopharyngeal swab samples in Polymerase Chain Reaction (PCR) analysis as the reference standard in COVID-19 diagnosis were included in the present study. The diagnostic criteria of the World Health Organization's (WHO) interim guidance were used [18]. All consecutive COVID-19 patients who were admitted to Ankara City Hospital from March 15th, 2021 to April 30th, 2022, were enrolled (ACH Cohort). Two-time frames were used, based on the COVID-19 pandemic and Omicron variant declaration date of WHO, and defined by the date of COVID-19 diagnosis: pre-Omicron era (March 15th, 2021–November 15th, 2022) and Omicron era (December 15th, 2022–April 30th, 2022). These two time frames were used to split the patients into two groups. The patients' data were obtained from Infectious Diseases Clinics and Intensive Care Units of Ankara City Hospital, a 4190-bed academic medical center in Ankara, Turkey. Demographic, clinical, and laboratory data were gathered from hospital and laboratory information systems (HIS and LIS) electronic medical records and case record forms. Only initial laboratory results of the patients within 72 h of admission were recorded. The severity of the patients included in the study was analyzed using the same criteria of the algorithm to examine the concordance of the existing algorithm with real-world data.

To examine the concordance of the AI algorithm with real-world data, a previously established and externally validated deep learning approach for predicting the severity of COVID-19 patients using a sparse set of laboratory markers was applied [16]. In this study, a deep neural network-trained model was used to predict clinical outcomes using different cohorts of outcome-matched patient data from four COVID-19 epicenters. A number of experiments were carried out to investigate the contributions of the different data inputs to the assigned surrogate outcomes and to gain a better understanding of the redundancies by minimising the interactions between the input parameters. Finally, a predictive tool that can maintain accuracy with limited input data was developed. Comparisons with other methods in the literature were made on the performance of the proposed models.This predictive algorithm was a trained and tested AI-based network using COVID-19 patient records from three healthcare systems from the US and one from Spain (Development Training Cohort and Testing Cohort). The model provides a severity risk score along with the likelihood of various clinical outcomes, namely ventilator use, end-organ failure and mortality. The predictor laboratory tests included in the algorithm are standard blood tests, namely creatinine, CRP, D-dimer, eosinophil (%), ferritin, INR, LDH, lymphocyte (%), and Troponin I, and they were routinely measured in laboratory features for COVID-19 patients in Ankara City Hospital. Prognostic and predictive parameters obtained as a result of the algorithm and compared were disease severity (0–4), ventilator use, end-organ failure, and risk of mortality within 30 days of hospital admission.

### 2.1 Selection of participants

In our hospital, oro-nasopharyngeal swab samples for RT-PCR were obtained for all suspected patients in addition to routine blood tests during the pandemic period (after March 2020). Physicians of this study at the Infectious Diseases Clinic identified COVID-19 using consistent clinical signs, such as fever and respiratory symptoms, evidence of pneumonia on computerized tomography (CT), and/or positive SARS-CoV-2 PCR results in accordance with the WHO interim guidance [18]. The study included patients who required intensive care when admitted or at any time during their hospital stay, as well as those who did not require ICU care. Patients who died within the first 24 h following their hospital admission were excluded from the study. The additional exclusion criteria are not having a positive SARS-CoV-2 PCR result, being pregnant, being under the age of 18, not being a case from the determined time frames, and not having laboratory results included in the model within 72 h of admission. The data of COVID-19 patients whose laboratory tests were requested and carried out within 72 h of hospital admission were retrospectively examined and analyzed.

### 2.2 Clinical laboratory analyses

Analyzers performed all laboratory parameters following the manufacturer's instructions. All reagents, controls and calibrators for laboratory parameter measurements were obtained from Siemens Healthcare Diagnostics (Erlangen, Germany). All devices: Atellica Solution Immunoassay and Clinical Chemistry Analyzer (Siemens Healthcare Diagnostics, Erlangen, Germany) for serum creatinine, CRP, ferritin, LDH, and Troponin I; the ADVIA 2120 Hematology System (Siemens Healthcare Diagnostics, Erlangen, Germany) for eosinophil (%) and lymphocyte (%); and Sysmex CS-5100 System (Siemens Healthcare Diagnostics, Erlangen, Germany) for D-dimer and INR, were employed in accordance with Westgard's quality control rules. The following methods were used for clinical chemistry and immunoassay tests: the kinetic alkaline picrate method for creatinine, the lactate to pyruvate forward reaction method for LDH, and the immunoturbidimetric methods for CRP, chemiluminometric assay for ferritin and Troponin I.

According to a study by Ricos C. et al., the acceptable total analytical error (TEa) was computed using data from internal and external quality control for creatinine, CRP, D-dimer, eosinophil (%), ferritin, INR, LDH, lymphocyte (%), and Troponin I and tests and the values of 3.61 %, 7.20 %, 6.50 %, 5.72 %, 11.2 %, 0.2 %, 3.84 % 5.72 % and 5.30 % were under the TEa limit of allowable error based on specifications on Westgard's website; ±8.87 %, ±56.6 %, ±28 %, ±37.1 %, ±16.9 %, ±5.3 %, ±11.4 %, ±17.6 % and ±27.9 %, respectively [19].

### 2.3 Statistical analysis

Performance evaluation was conducted in a real-world data cohort. For the retrospectively obtained data, descriptive statistics were generated, and the distribution of the data was examined. The log-rank test and Kaplan-Meier survival analysis were used to assess the performance of the DL algorithm for prognostic analysis. In addition, the compatibility between the prognostic data predicted by the DL algorithm and the actual clinical course before and after the Omicron variant were evaluated by creating different models with logistic regression analysis. Additionally, sensitivity, specificity, and positive and negative predictive values were determined using ROC analysis for each data's separate cut-off values. Statistical methods were presented quantitatively and visually using the SPSS (IBM version 26.0) program. Statistical significance was defined as P < 0.05.

## 3 Results

### 3.1 Clinical, demographic characteristics and laboratory features

A total of 7028 and 3554 patients, making up the data sets for the development training cohort and the development testing cohort, respectively, were recruited from the USA. The study included 638 hospitalized patients (ACH cohort) with COVID-19 diagnosis according to WHO criteria (13). In addition to clinical symptoms and findings consistent with COVID-19, all patients had PCR confirmation. All SARS CoV-2 PCR-positive patients were grouped based on severity scores of the algorithm as Severity 0, Severity 1, Severity 2, Severity 3, and Severity 4. The description of severity levels, criteria, and prevalence in DL data sets are shown in Table 1, and the distributions of the predictor laboratory tests in the sensitivity analysis of the cohort data set are shown in Table 2. The severity scores were assigned based on the worst condition of the patient during the course of the hospital stay on an ordinal scale from 0 to 4. The definitions of the various scales were primarily based on Berlin criteria and Sequential Organ Failure Assessment (SOFA) scores [20,21]. A total of 10 variables comprised of laboratory tests and demographics for patients in DL data sets were depicted in Table 3. The table shows the statistical significance (p-value) of the difference in the mean values between the sub-groups with mortality and those with discharge. All the p-values of less than 0.05 indicate the potential of the variable to be an independent predictor of mortality. As shown, while all variables were significant independent predictors for mortality in the pre-Omicron group except INR, in the Omicron group, creatinine, INR, LDH, % lymphocytes and Troponin I were significant independent predictors.

**Table 1:** Description, criteria, and prevalence of severity levels in data sets.

	Severity 0	Severity 1	Severity 2	Severity 3	Severity 4
Description	No respiratory problem	Mild respiratory problem	Moderate to severe respiratory problem	Severe respiratory problem with organ damage	Mortality within 30 days of admission
Criteria	No O_2_ supplement required	Hypoxic patients requiring O_2_ supplement	Hypoxic patients requiring high flow nasal cannula, BIPAP, or invasive O_2_ supplement therapy	Same as severity 2 along with an increase in SOFA score (renal, liver) by 2 and/or renal replacement therapy	In-hospital mortality within 30 days or transfer to hospice
Prevalence per data set (n: observed severity/total patients)					
Development Cohort-PreOmicron Era	3103/10582	4887/10582	741/10582	470/10582	1381/10582
ACH Cohort-PreOmicron + Omicron Era	189/638	152/638	100/638	59/638	138/638
ACH Cohort-PreOmicron Era	99/366	96/366	61/366	35/366	75/366
ACH Cohort-Omicron Era	90/272	56/272	39/272	24/272	63/272

Legend: n, number of cases.

**Table 2:** The features included in the algorithm of the ACH Data Set.

Characteristics	Severity 0	Severity 1	Severity 2	Severity 3	Severity 4
Age	48 (46, 51) (189)	63 (61, 66) (152)	61 (58, 65) (100)	66 (62, 71) (59)	76 (74, 78) (138)
Creatinine (mg/dL)	0.9 (0.8, 0.9) (189)	0.9 (0.9, 1.0) (152)	1.0 (0.9, 1.1) (100)	1.4 (1.2, 1.6) (59)	1.4 (1.3, 1.6) (138)
CRP (mg/L)	35 (28, 42) (189)	90 (80, 100) (152)	132 (117, 146) (100)	115 (96, 134) (59)	124 (112, 136) (138)
D-Dimer (mg/L)	1.1 (0.7, 1.4) (182)	1.6 (1.1, 2.1) (151)	3.4 (2.1, 4.8) (100)	3.7 (2.0, 5.4) (58)	3.6 (2.7, 4.5) (137)
Eosinophil %	1.5 (1.3, 1.8) (189)	0.8 (0.6, 0.9) (152)	0.3 (0.2, 0.3) (100)	0.6 (0.1, 1.1) (59)	0.5 (0.4, 0.6) (138)
Ferritin (μg/L)	142 (116, 168) (175)	488 (391, 584) (151)	657 (525, 789) (100)	1000 (327, 1673) (59)	539 (430, 649) (138)
INR	1.1 (1.1, 1.1) (187)	1.2 (1.1, 1.2) (150)	1.1 (1.1, 1.2) (100)	1.3 (1.1, 1.4) (58)	1.3 (1.2, 1.4) (138)
LDH (U/L)	250 (240, 261) (187)	357 (337, 378) (152)	498 (459, 537) (100)	524 (412, 637) (59)	446 (415, 477) (138)
Lymphocytes %	25 (23, 27) (189)	16 (14, 17) (152)	13 (10, 16) (100)	11 (9, 13) (59)	11 (10, 13) (138)
Troponin I (ng/L)	16 (4, 28) (164)	35 (15, 55) (147)	132 (38, 227) (97)	670 (111, 1229) (57)	401 (189, 612) (135)

Legend: mean (confidence interval low, high) (count).

**Table 3:** Cohort statistics of laboratory variables included in the algorithm.

	Development Training Cohort- PreOmicron Era	Development Testing Cohort- PreOmicron Era	Mortality p-value	ACH Cohort-PreOmicron + Omicron Era	Mortality p-value	ACH Cohort-PreOmicron Era	Mortality p-value	ACH Cohort-Omicron Era	Mortality p-value
Age	60.5 (60.0, 60.9) (7026)	58.3 (57.6, 58.9) (3551)	<0.001	62 (60, 63) (638)	0.050	59 (57, 61) (366)	0.04	65 (63, 68) (272)	0.474
Creatinine (mg/dL)	1.4 (1.4, 1.5) (6745)	1.3 (1.3, 1.4) (3255)	0.892	1.1 (1.0, 1.1) (638)	<0.001	1.0 (1.0, 1.1) (366)	<0.001	1.1 (1.0, 1.2) (272)	0.015
CRP (mg/L)	111 (107, 115) (1797)	107 (102, 113) (991)	0.087	90 (84, 96) (638)	0.007	93 (85, 100) (366)	0.002	86 (76, 95) (272)	0.614
D-Dimer (mg/L)	2.6 (2.3, 2.9) (3077)	2.8 (2.4, 3.2) (1913)	0.798	2.4 (2.0, 2.8) (628)	0.029	2.1 (1.6, 2.6) (363)	0.002	2.8 (2.1, 3.4) (265)	0.917
Eosinophil %	0.6 (0.6, 0.7) (5886)	0.8 (0.8, 0.9) (2811)	0.787	0.8 (0.7, 1.0) (638)	0.154	0.7 (0.6, 0.8) (366)	0.002	1.0 (0.8, 1.2) (272)	0.593
Ferritin (μg/L)	1183 (842, 1523) (1814)	774 (629, 919) (1622)	0.222	478 (400, 556) (623)	<0.001	523 (400, 646) (362)	<0.001	415 (343, 488) (261)	0.136
INR	1.2 (1.2, 1.2) (4512)	1.2 (1.2, 1.3) (2292)	0.492	1.2 (1.1, 1.2) (633)	0.101	1.2 (1.1, 1.2) (364)	0.997	1.2 (1.1, 1.2) (269)	0.007
LDH (U/L)	363 (357, 370) (3334)	341 (331, 351) (1718)	0.387	383 (366, 400) (636)	<0.001	403 (383, 423) (366)	<0.001	355 (327, 384) (270)	<0.001
Lymphocytes %	17 (17, 18) (6415)	18 (18, 18) (3162)	0.071	17 (16, 18) (638)	<0.001	16 (15, 18) (366)	0.001	17 (15, 18) (272)	0.029
Troponin I (ng/L)	198 (109, 286) (3581)	142 (78, 207) (2034)	0.913	188 (113, 263) (600)	<0.001	174 (67, 280) (347)	0.002	208 (107, 309) (253)	0.007

Legend: mean (confidence interval low, high) (number of patients for whom the value was recorded.

### 3.2 Performance of individual features and model performance in the testing data set

In the ACH cohort, of 638 eligible COVID-19 patients, 366 patients were grouped in the pre-Omicron variant pandemic time frame, and 272 patients were grouped in the Omicron variant pandemic time frame; and named as pre-Omicron group and Omicron group, respectively.

Table 4 summarises feature performance characteristics in the development and ACH cohorts, stratified according to Omicron pandemic status. PVs of 0.78 or higher were observed for severe patients in both the pre-Omicron and Omicron periods. This is consistent with algorithm performance in the development cohort.

**Table 4:** Performance characteristics of AI data sets.

	Development Testing Cohort-PreOmicron Era			ACH Cohort-PreOmicron + Omicron Era			ACH Cohort-PreOmicron Era			ACH Cohort-Omicron Era		
	Sev ≥ 2	Sev ≥ 3	Sev = 4	Sev ≥ 2	Sev ≥ 3	Sev = 4	Sev ≥ 2	Sev ≥ 3	Sev = 4	Sev ≥ 2	Sev ≥ 3	Sev = 4
Sensitivity	0.48 (0.47,0.49)	0.39 (0.38,0.40)	0.33 (0.32,0.36)	0.84 (0.84,0.84)	0.81 (0.81,0.81)	0.75 (0.75,0.76)	0.81 (0.81,0.81)	0.79 (0.79,0.80)	0.68 (0.68,0.69)	0.89 (0.88,0.89)	0.83 (0.83,0.84)	0.84 (0.84,0.85)
Specificity	0.85 (0.85,0.86)	0.92 (0.92,0.93)	0.97 (0.96,0.97)	0.70 (0.70,0.71)	0.66 (0.65,0.66)	0.71 (0.71,0.71)	0.73 (0.73,0.73)	0.67 (0.67,0.67)	0.72 (0.71,0.72)	0.67 (0.66,0.67)	0.64 (0.63,0.64)	0.70 (0.69,0.70)
PPV	0.48 (0.47,0.49)	0.48 (0.47,0.48)	0.55 (0.54,0.56)	0.77 (0.77,0.78)	0.69 (0.69,0.69)	0.62 (0.62,0.63)	0.77 (0.76,0.77)	0.68 (0.68,0.68)	0.58 (0.58,0.58)	0.79 (0.79,0.79)	0.71 (0.71,0.71)	0.67 (0.67,0.68)
NPV	0.87 (0.86,0.87)	0.90 (0.9,0.9)	0.93 (0.92,0.94)	0.78 (0.78,0.78)	0.78 (0.78,0.79)	0.82 (0.82,0.82)	0.78 (0.77,0.78)	0.79 (0.78,0.79)	0.80 (0.79,0.80)	0.80 (0.80,0.81)	0.78 (0.78,0.79)	0.86 (0.85,0.86)

Legend: mean (confidence interval low, high); PPV: Positive Predictive Value; NPV: Negative Predictive Value.

**Fig. 1:**
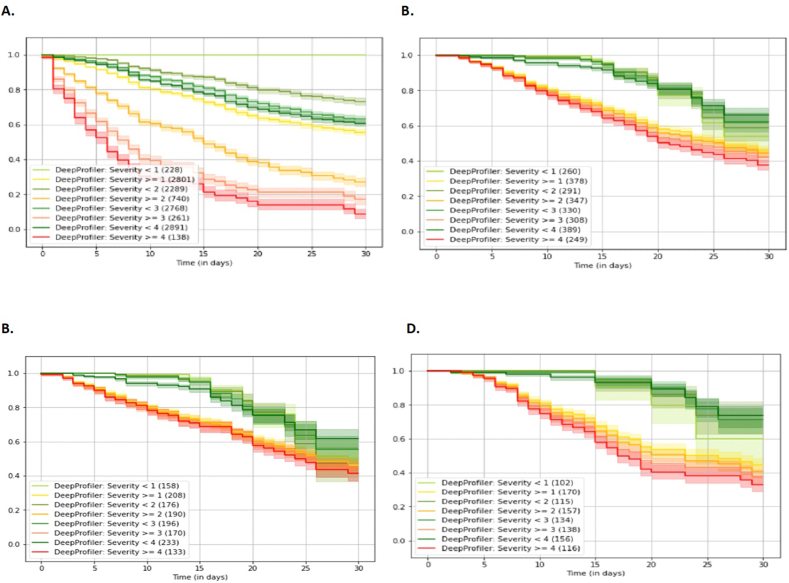
The Kaplan-Meier Statistics of DL data sets. Kaplan-Meier curves comparing the shaded regions identify the 95 % confidence interval. A. The Kaplan-Meier Statistics of The Development Cohort Testing Data Set; B. The Kaplan-Meier Statistics of the ACH Cohort Data Set- PreOmicron + Omicron Era C. The Kaplan-Meier Statistics of the ACH Cohort Data Set- PreOmicron Era; D. The Kaplan-Meier Statistics of the ACH Cohort Data Set- Omicron Era.

Fig. 1 depicts the Kaplan-Meier curve in the developing cohort and in total and subgroups of the ACH cohort data set, which contains COVID-19 patients with PCR-confirmed positive (no PCR negatives). The concordance index values, which were computed based on the predicted severity scores and ground truth values based on patient outcome [22], were 0.71, 0.80, 0.79, and 0.81 for the development testing cohort data set-pre-Omicron era, ACH data set-pre-Omicron + Omicron era, ACH data set-pre-Omicron Era, and ACH data set- Omicron era, respectively. The Kaplan-Meier curves were presented for predicted cohorts as well as patient disease severity (Severity 0–4) in all data sets. Kaplan-Meier curves showed a clear separation between the low vs. high severity levels for all time frames of COVID-19 (Fig. 1(A–D)).

**Fig. 2:**
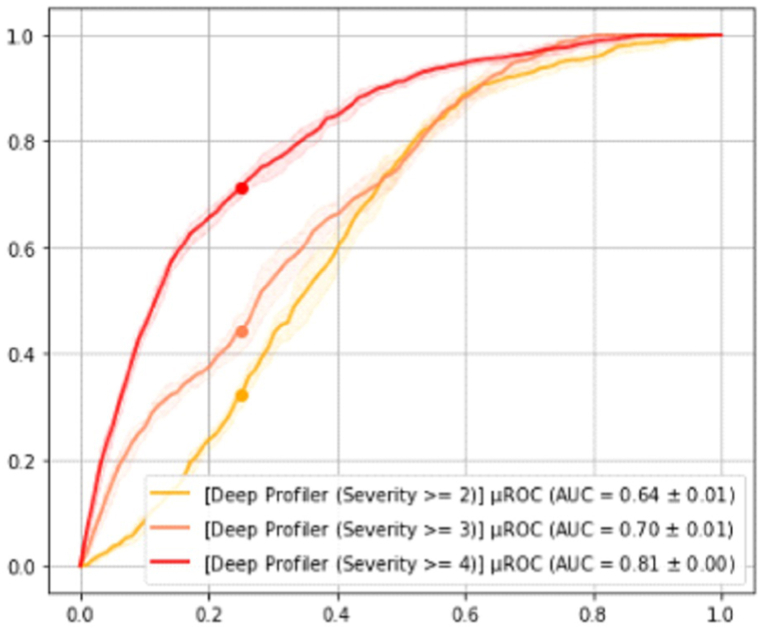
The ROC curve of the ACH cohort data set.

In Fig. 2, the AUC was presented in the ACH cohort data set. AUCs ranged from 0.64 ± 0.01 to 0.81. Analysis showed consistent concordance among the pre-Omicron and Omicron cases.

**Fig. 3:**
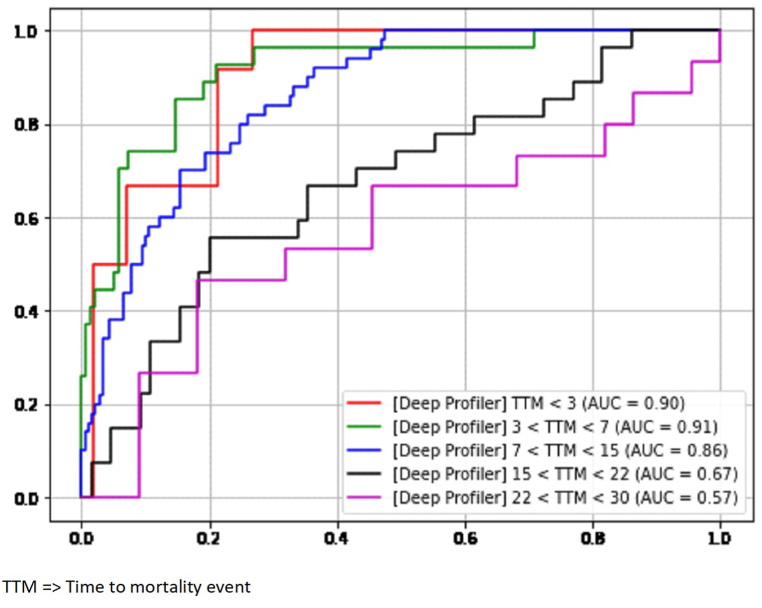
The ROC curve of time to mortality Event of the ACH cohort data set.

Fig. 3 depicts the ROC for time to mortality at 3, 7, 15, 22, and 30 days from admission. AUCs ranged from 0.57 to 0.90. Analysis showed consistent concordance among the pre-Omicron and Omicron cases. The accuracy of prediction is higher for patients who are likely to experience higher levels of severity within the first three days and 3–7 days after admission, with AUC values of 0.90 and 0.91 respectively.

With respect to the predictive accuracy of mortality (severity level = 4), the DL algorithm's negative predictive value (NPV) was 0.80 (Sensitivity: 0.68 and Specificity: 0.72), 0.86 (Sensitivity: 0.84 and Specificity: 0.70) for pre-Omicron and Omicron variants, respectively. According to these results, the previously developed and validated DL algorithm is useable and generalizable for both mortality or severity predictions for Omicron or pre-Omicron variants and guides clinicians in the predictability of disease outcomes.

## 4 Discussion

To our knowledge, this is the first study that demonstrates the previously developed and validated DL algorithm utilizing age and nine laboratory indicators accurately predict severity in patients with COVID-19 both in the pre-Omicron phase and Omicron periods [16]. Although there are studies on the AI topic of COVID-19 patients performed in Turkey, none of them has compared different variant periods [23,24].

During the COVID-19 outbreak, the need for rapid identification and stratification of patients for supportive clinical care became urgent because of the heavy workload at the beginning of the outbreak. So far, many studies about AI use in COVID-19 using different approaches have been published. Different models, including the use of demographics, radiological data, symptomatology, and laboratory features with different obtained AUCs, have been reported. From the variables used in the models, symptomatology might not be obtained with structured, validated questionnaires, and outside of a research environment, it might be challenging to record these symptoms reliably. Furthermore, because they are not structurally recorded in modern medical records, these symptoms cannot be incorporated into automated risk assessments. Structured data collected during the clinical examination are the easiest to integrate and may have the least amount of variability from one institution to another. These include vital signs, demographic data, laboratory results, and radiological images. It is important to note that another model developed from chest computed tomography images had an AUC of 0.994 for the discrimination of COVID-19 from atypical or viral pneumonia [25]. However, due to the increased risk of infection spread from additional visits to radiology suites, national organizations advise against using radiological imaging for the diagnosis of COVID-19. Gülbay et al. from Turkey have demonstrated that a machine learning algorithm made of clinical and DL-segmentation-based radiological criteria, trained with a balanced data set, can successfully predict COVID-19 patients who may need intensive care [24]. Radiological image data might be very useful in models. However, they can be hard to find and have high costs in clinical settings, especially in low-capacity hospitals and in low-income countries. These approaches are also unlikely to be quickly implemented due to the difficulties in conducting optional radiological examinations during this pandemic.

The models, which are easy-to-use and based on the few numbers of routinely measured laboratory markers, could be more practical than the others and thus more widely used. The model used in this study includes nine blood biomarkers and age, which capture a range of underlying biological processes known to be early independent predictors of disease severity. These processes include immune response (i.e., lymphocytes and eosinophils), kidney and liver function (i.e., creatinine and LDH), cardiac function (i.e., Troponin I), inflammation processes (i.e., CRP and ferritin), and coagulation process (i.e., D-dimer and INR) [16]. The model includes standard laboratory tests that are widely available with rapid turnaround time. The main time limitation is related to phlebotomy and sample processing, as the machine learning model can be completed almost immediately. Also, all these parameters were carried out routinely throughout the management of COVID-19; thus, we can easily compare the results obtained with the algorithm by leveraging a retrospective cohort. Previous studies have explored the use of laboratory data, along with non-radiological structured clinical data or demographics, in the diagnosis of COVID-19 through machine learning techniques.

On November 11, 2021, a sequenced Omicron case was first reported from Botswana. A few days later, a traveler from South Africa was also reported from Hong Kong with a sequenced Omicron case [26]. Concerns over the potential effects of this novel VoC on clinical presentation have grown since then. Febrile children with COVID-19 have a much greater incidence of seizures, which has led to an increase in hospitalizations, especially in children younger than five years in the Omicron era than in the pre-Omicron era [27,28]. In adults, the Omicron period was independently associated with a lower risk of inpatient mortality [29]. Therefore, we found fewer variables to be predictors of mortality in the Omicron period than in the pre-Omicron period. In the present study, the concordance index values were 0.71, 0.80, 0.80, and 0.81 for the development cohort data set, total testing data set, pre-Omicron testing data set, and Omicron testing data set, respectively. Although outcomes of COVID-19 inpatients evolved throughout the pandemic and were affected by changing virus variants, the DL algorithm remained valid for predicting outcomes for hospitalized patients who were admitted to hospitals with different disease severity. The algorithm using age and frequently measured laboratory parameters continues to estimate the disease's severity accurately.

COVID-19 outcomes among hospitalized patients may have changed due to new variants, therapies and vaccine availability. However, the previously developed and externally validated deep learning algorithm is still valid in the era of the Omicron variant, accurately predicting disease severity [16]. Because the DL algorithm uses blood markers, different therapies may be expressed in these markers, which reflect the physiological response to vaccination-induced immunity. The model only includes the characteristics of the blood test performed as a routine part of the hospital visit. Furthermore, the fact that these tests came from several hospitals suggests that the model is resistant to variations in specimen collection, handling, and instrumentation. This demonstrates the model's adaptability to institutions using various specimen handling and laboratory processing techniques. To further describe the model's performance and its therapeutic applicability in efficiently managing a focused, particularly immunized patient population, a prospective validation study is required.

Chieregato et al. recommended a severity prediction model that combines machine learning and deep learning as an adjunct to patient risk assessment in clinical practice [30]. The authors deemed their model more suitable for predicting the severity of ICU admission outcomes in a clinical setting rather than mortality. In this study, we concluded that the previously developed and validated DL algorithm is useable for both mortality or severity predictions for Omicron or pre-Omicron variants and guides clinicians in the predictability of disease outcomes [16].

According to Chi et al., their study found that a deep learning model was able to accurately predict short-term mortality or the outcome of hospice care on the second day of admission in the general inpatient population [31]. We also showed that both pre-Omicron and Omicron prediction accuracy was higher for potentially more severe patients in the first 3 days and 3–7 days post-admission.

In another study performed by our group, the concordance index values (CI) of the predicted severity levels on the internal testing dataset and external validation dataset were 0.71 and 0.64, respectively [16]. In the present study, concordance index values were 0.80, 0.79, and 0.81 for the ACH data set-pre-Omicron + Omicron era, ACH data set-pre-Omicron Era, and ACH data set- Omicron era, respectively. Wang et al. conducted a study in which deep learning models were utilized to predict the progression of COVID-19 patients to critical disease, using baseline CT and clinical data. A concordance index value of 0.80 was attained by the models [32]. Cheng et al. demonstrated that the inclusion of imaging improves the clinical-only model significantly. This resulted in an increase in the AUC from 0.653 to 0.727 (p = 0.039) and the accuracy from 0.657 to 0.732 [11]. Singh et al. performed external validation of the model using a publicly available Mt. Sinai dataset, achieving a performance of AUC of 0.74 + 0.01 to predict mortality [16]. The prediction accuracy of severity 4 (i.e., mortality) has an AUC of 0.81 for the full model using ten parameters in this study, and imaging was not included in the deep learning model. However, although we have included different variants of COVID-19, our concordance index values and AUCs were similar to those of these studies.

Some of the limitations are as follows: First, only one DL algorithm was evaluated, and this model's performance was not compared with other machine learning models. Second, the dataset comes from a single center. However, while this provides us with data homogeneity, it caused a decrease in the generalization power of this algorithm. The study examined the issue of missing values and outliers, in addition to the limited size of the dataset that was employed. Another limitation of the study is that we have not taken into account the vaccination status, therapies of patients or comorbidities. However, just like the variants, the vaccination statuses and medications could have modulated the markers so that the model could predict the severity without needing to have information about the vaccination statuses or medications.

## 5 Conclusions

Throughout the pandemic, outcomes for COVID-19 inpatients changed due to shifting demographics, novel viral strains, and vaccination. Based on our findings, age and blood test results routinely measured on admission are valid to predict disease severity both in the pre-omicron and omicron era. A machine learning model that incorporates frequently used and simple-to-use laboratory markers with discriminatory accuracy can be utilized as a clinical decision-support tool to aid physicians in making clinical judgments for patients hospitalized with COVID-19.

## Ethical statement

This retrospective study was approved by the ethics board of Ankara City Hospital (No. E1-22-2442).

## Data availability statement

The data includes patient information and hence cannot be made publicly available.

## CRediT authorship contribution statement

**Gulsen Yilmaz:** Writing – original draft. **Sevilay Sezer:** Writing – review & editing. **Aliye Bastug:** Data curation. **Vivek Singh:** Methodology. **Raj Gopalan:** Methodology. **Omer Aydos:** Data curation. **Busra Yuce Ozturk:** Data curation. **Derya Gokcinar:** Data curation. **Ali Kamen:** Writing – review & editing. **Jamie Gramz:** Writing – review & editing. **Hurrem Bodur:** Supervision. **Filiz Akbiyik:** Supervision, Project administration.

## Declaration of competing interest

The authors declare the following financial interests/personal relationships which may be considered as potential competing interests:

V.S., R. G., A.K. J.G. and F.A. are employees of Siemens Healthineers.

## References

[1] Organization Who (2022). 26 August. https://covid19.who.int/.

[2] Yang Y., Peng F., Wang R., Guan K., Jiang T., Xu G. (2020). The deadly coronaviruses: the 2003 SARS pandemic and the 2020 novel coronavirus epidemic in China. J. Autoimmun..

[3] Henry B.M., De Oliveira M.H.S., Benoit S., Plebani M., Lippi G. (2020). Hematologic, biochemical and immune biomarker abnormalities associated with severe illness and mortality in coronavirus disease 2019 (COVID-19): a meta-analysis. Clin. Chem. Lab. Med..

[4] Abd-Alrazaq A., Alajlani M., Alhuwail D., Schneider J., Al-Kuwari S., Shah Z. (2020). Artificial intelligence in the fight against COVID-19: scoping review. J. Med. Internet Res..

[5] Chen J., See K.C. (2020). Artificial intelligence for COVID-19: rapid review. J. Med. Internet Res..

[6] Moulaei K., Shanbehzadeh M., Mohammadi-Taghiabad Z. (2022). Comparing machine learning algorithms for predicting COVID-19 mortality. BMC Med. Inf. Decis. Making.

[7] Shanbehzadeh Mostafa, Nopour Raoof, Kazemi-Arpanahi Hadi (2022). Using decision tree algorithms for estimating ICU admission of COVID-19 patients. Inform. Med. Unlocked.

[8] Organization WH (2021). 28 November. https://www.who.int/news/item/28-11-2021-update-on-omicron.

[9] Ghaderzadeh M., Eshraghi M.A., Asadi F., Hosseini A., Jafari R., Bashash D., Abolghasemi H. (2022 Apr 21). Efficient framework for detection of COVID-19 omicron and Delta variants based on two intelligent phases of CNN models. Comput. Math. Methods Med..

[10] Abd-Alrazaq A., Schneider J., Alhuwail D., Hamdi M., Al-Kuwari S., Al-Thani D. (2021). Artificial intelligence in the fight against the COVID-19 pandemic: opportunities and challenges. Multiple Perspectives on Artificial Intelligence in Healthcare.

[11] Cheng J., Sollee J., Hsieh C., Yue H., Vandal N., Shanahan J. (2022). COVID-19 mortality prediction in the intensive care unit with deep learning based on longitudinal chest X-rays and clinical data. Eur. Radiol..

[12] Heidari A., Jafari Navimipour N., Unal M. (2022). Machine learning applications for COVID-19 outbreak management. Neural Comput. Appl..

[13] Bacanin Nebojsa, Venkatachalam K. (2023). Timea Bezdan, Miodrag Zivkovic, Mohamed Abouhawwash, A novel firefly algorithm approach for efficient feature selection with COVID-19 dataset. Microprocess. Microsyst..

[14] Khan M., Mehran M.T., Haq Z.U., Ullah Z., Naqvi S.R., Ihsan M. (2021). Applications of artificial intelligence in COVID-19 pandemic: a comprehensive review. Expert Syst. Appl..

[15] Khan I.U., Aslam N., Aljabri M., Aljameel S.S., Kamaleldin M.M.A., Alshamrani F.M. (2021). Computational intelligence-based model for mortality rate prediction in COVID-19 patients. Int. J. Environ. Res. Publ. Health.

[16] Singh V., Kamaleswaran R., Chalfin D., Buño-Soto A., San Roman J., Rojas-Kenney E. (2021). A deep learning approach for predicting severity of COVID-19 patients using a parsimonious set of laboratory markers. iScience.

[17] Lou B., Doken S., Zhuang T., Wingerter D., Gidwani M., Mistry N. (2019). An image-based deep learning framework for individualising radiotherapy dose: a retrospective analysis of outcome prediction. The Lancet Digital Health.

[18] Organization WH (2020). Clinical management of severe acute respiratory infection (SARI) when COVID-19 disease is suspected: interim guidance. https://apps.who.int/iris/handle/10665/331446.

[19] (2020). Desirable Specifications for Total Error. https://www.westgard.com/biodatabase1.htm.

[20] Ferguson N.D., Fan E., Camporota L., Antonelli M., Anzueto A., Beale R. (2012). The Berlin definition of ARDS: an expanded rationale, justification, and supplementary material. Intensive Care Med..

[21] Lambden S., Laterre P.F., Levy M.M., Francois B. (2019). The SOFA score—development, utility and challenges of accurate assessment in clinical trials. Crit. Care.

[22] 28 June 2022 [Available from: https://lifelines.readthedocs.io/en/latest/lifelines.utils.html#lifelines.utils.concordance_index.

[23] Çubukçu H.C., Topcu D.İ., Bayraktar N., Gülşen M., Sarı N., Arslan A.H. (2022). Detection of COVID-19 by machine learning using routine laboratory tests. Am. J. Clin. Pathol..

[24] Gülbay M., Baştuğ A., Özkan E., Öztürk B.Y., Mendi B.A.R., Bodur H. (2022). Evaluation of the models generated from clinical features and deep learning-based segmentations: can thoracic CT on admission help us to predict hospitalized COVID-19 patients who will require intensive care?. BMC Med. Imag..

[25] Ardakani A.A., Kanafi A.R., Acharya U.R., Khadem N., Mohammadi A. (2020). Application of deep learning technique to manage COVID-19 in routine clinical practice using CT images: results of 10 convolutional neural networks. Comput. Biol. Med..

[26] Karim S.S.A., Karim Q.A. (2021). Omicron SARS-CoV-2 variant: a new chapter in the COVID-19 pandemic. Lancet.

[27] Iijima H., Kubota M., Ogimi C. (2022). Change in seizure incidence in febrile children with COVID-19 in the era of omicron variant of concern. Journal of the Pediatric Infectious Diseases Society.

[28] Setiabudi D., Sribudiani Y., Hermawan K., Andriyoko B., Nataprawira H.M. (2022). The Omicron variant of concern: the genomics, diagnostics, and clinical characteristics in children. Frontiers in Pediatrics.

[29] Stepanova M., Lam B., Younossi E., Felix S., Ziayee M., Price J. (2022). The impact of variants and vaccination on the mortality and resource utilization of hospitalized patients with COVID-19. BMC Infect. Dis..

[30] Chieregato M., Frangiamore F., Morassi M., Baresi C., Nici S., Bassetti C. (2022). A hybrid machine learning/deep learning COVID-19 severity predictive model from CT images and clinical data. Sci. Rep..

[31] Chi S., Guo A., Heard K., Kim S., Foraker R., White P. (2022). Development and structure of an accurate machine learning algorithm to predict inpatient mortality and hospice outcomes in the coronavirus disease 2019 era. Med. Care.

[32] Wang R., Jiao Z., Yang L., Choi J.W., Xiong Z., Halsey K. (2022). Artificial intelligence for prediction of COVID-19 progression using CT imaging and clinical data. Eur. Radiol..

